# Neurocysticercosis in Northern Peru: Qualitative Insights from men and women about living with seizures

**DOI:** 10.1371/journal.pntd.0008715

**Published:** 2020-10-09

**Authors:** Maria Amalia Pesantes, Luz Maria Moyano, Claire Sommerville, on behalf of Cysticercosis Working Group in Peru and COHESION Project

**Affiliations:** 1 CRONICAS, Centre of Excellence in Chronic Conditions, Universidad Peruana Cayetano Heredia, Miraflores, Lima, Peru; 2 Center for Global Health, Tumbes Facilities, Universidad Peruana Cayetano Heredia, Puerto el Cura Pizarro, Tumbes, Peru; 3 Gender Centre, Graduate Institute of International and Development Studies, Switzerland; Centro de Pesquisa Gonçalo Moniz-FIOCRUZ/BA, BRAZIL

## Abstract

**Background:**

Neurocysticercosis (NCC) is a helminthic disease of the central nervous system, and it is one of the leading causes of seizures and symptomatic epilepsy in countries with tropical regions like Peru. Studies of people with epilepsy in Peru’s northern coast have consistently found that between 30% and 50% of epilepsy cases is associated with NCC. There are few studies that report on the differences in incidence and prevalence of NCC by sex, and to our knowledge, none that consider the gendered dimensions of having epilepsy.

**Methodology:**

This qualitative study based on individual interviews (n = 9) and focus group discussions (n = 12) explored the challenges of diagnosis and the implications for everyday activities among men and women with epilepsy as well as the views of their family members on the impact of such condition.

**Principal findings:**

The explanatory models used by women to discuss their condition reflect low levels of decision-making power in areas such a reproductive health, health care access and treatment. For some women domestic violence is also a probable cause for seizures among women. The implications of living with neurocysticercosis and the accompanying seizures were reported differently by men and women. While women were mostly concerned about their capacity to perform their domestic responsibilities and their roles as mothers and caregivers; men were mostly concerned about the impact on their income generation activities. Women and men shared concern about the consequences of their condition on the wellbeing of their families.

**Conclusions/Significance:**

NCC is a disrupting experience for men and women in ways that reflect their position and roles in society: Women as caregivers within the home, men as income generators outside the home. Further gender research is needed to better understand and address the differential impacts of NCC and health system responses as well as gendered dimensions of prevalence and incidence. (268 words)

## Author summary

Neurocysticercosis (NCC) is a condition caused by a parasitic infection associated with tapeworms that usually results in seizures. NCC is common in tropical areas such as Northern Peru where families in rural communities breed pigs. Neurocysticercosis is a helminthic disease that is acquired through faecal-oral contamination. Poor sanitary facilities and poor hygienic practices are factors that increase the chances of infection.

NCC affects the central nervous system and this usually results in seizures and symptomatic epilepsy. In this article we explore the implications of living with NCC using a gender lens. We describe the different ways in which men and women explain and cope with seizures and show that while both men and women see their abilities to perform everyday tasks diminished; women’s concerns revolve around not being able to perform their responsibilities as mothers and housewives, while men worry about the wellbeing of the family as a result of their reduced capacity to engage in income generating activities. Further research to document whether gendered activities result in differential exposure to NCC is needed to have a better understanding of the various ways in which gender impacts the health status of men and women in poor rural areas.

## Introduction

Neurocysticercosis (NCC) is a helminthic disease of the central nervous system, and it is one of the leading causes of seizures and symptomatic epilepsy in rural areas of countries with tropical regions like Peru [1,2]. It occurs when the human central nervous system is infected by the vesicular stage of *Taenia solium tapeworm* [3]. Humans are the primary host and pigs are the intermediate host of *Taenia solium*. Pigs get infected by eating human faeces contaminated with the *Taenia solium* eggs or proglotides. This happens when pigs are reared close to where humans defecate [4].

**Fig 1:**
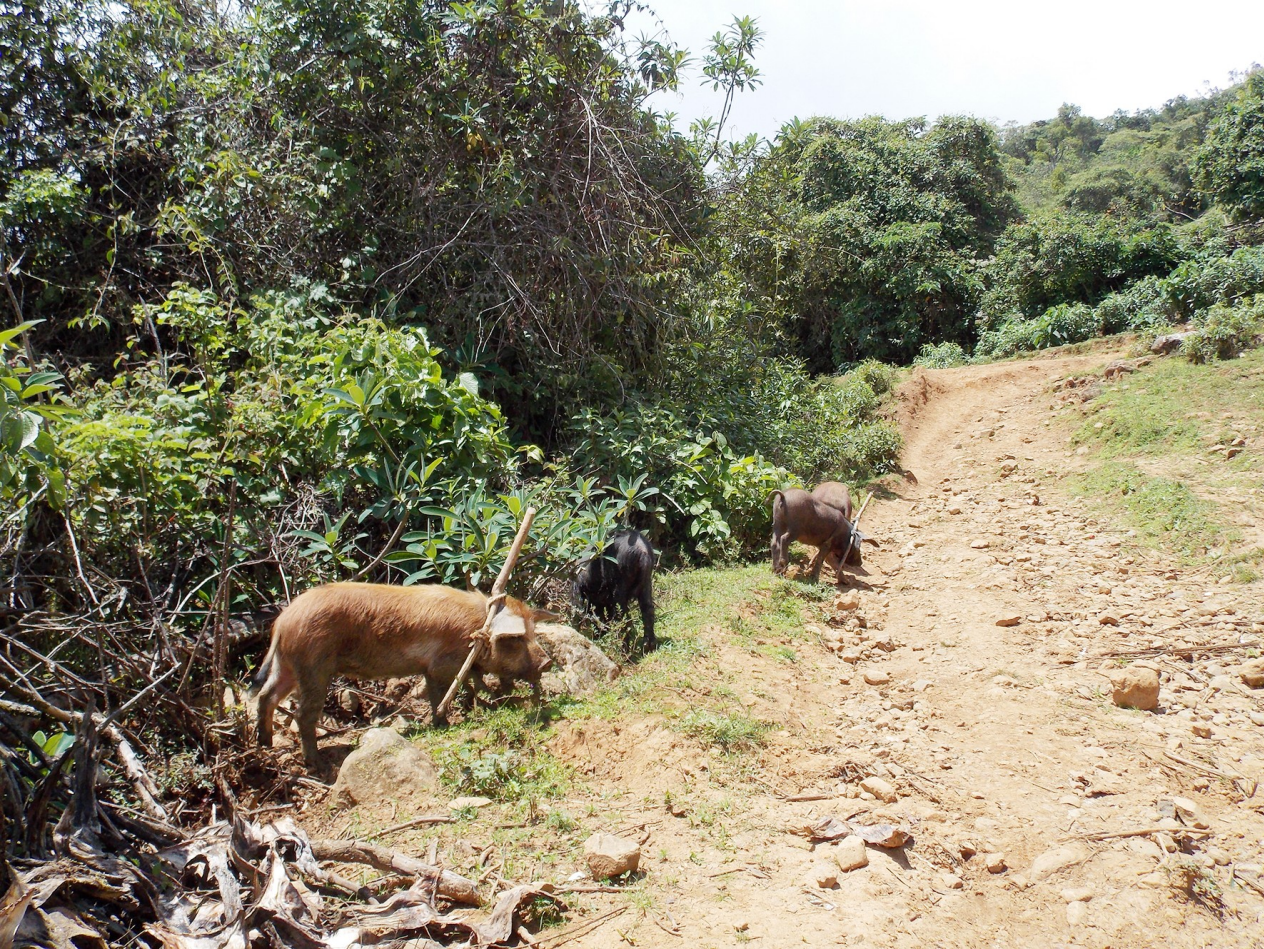
Pigs roaming freely in rural communities in northern Peru.

Pigs in rural communities in northern Peru are usually only corralled during the night. During the day they roam freely and are exposed to open human defecation areas, increasing the risk of contamination as seen in Fig 1 [5–7]. A study conducted in rural northern Peru found that more than 80% of free-range pigs roam within 100 metres of their owner’s homes, thus making it easy to identify a human tapeworm carrier if the pigs are found to be infected [8].

Human beings acquire NCC through faecal-oral contamination by ingesting parasite eggs from a human harboring an adult tapeworm in the intestines [10]. Individuals that live in areas with poor sanitation and with inadequate pig-raising practices are more likely to ingest the eggs of the *Taenia solium* [5,9]. However, scholars know little about whether women or men are more susceptible of *Taenia solium* because of the activities they perform. The risk factors associated with human taeniasis and NCC in areas like northern Peru include poor hygienic practices [4], eating poorly cooked pork and close proximity to a carrier of *Taenia solium* [4,7]. We do not know if there are differences in pork consumption between men and women but we know that there are differences in their role in raising pigs and their use of sanitation facilities [1]. A cohort study with 6439 participants in a similar region in northern Peru found that women practice open defecation 8.6 times less than men which appeared to be a protective factor against NCC.

There is wide variety of neurological expression and variability in the clinical forms of NCC, but all studies agree that seizures are the most common symptom, occurring in 60%–90% of patients [11]. Serologic or CT scans of people with epilepsy from Peru´s northern coast have consistently found that between 30% and 50% of epilepsy is associated with NCC [1,12,13]. When compared with industrialized countries, the prevalence and incidence of epilepsy in endemic areas of Peru is more than double [4]. The increase of tourism and migration toward endemic regions has made NCC a “neglected but not forgotten” disease [14].

While few studies have specifically examined differences between men and women in the prevalence of NCC, some have documented sex differences in the susceptibility and severity of this disease [5,15–18]. For example, a study in Ecuador among NCC hospitalized patients found the odds of having transitional cysts were 1.5-fold higher for female patients than for male patients, even after controlling for healthcare access [15]. Another study among patients recently diagnosed with NCC in Ecuador found that women reported more seizures than men [10]. These differences, according to researchers, could be explained by men and women’s different health-seeking behaviors: women seek care more frequently for less disruptive partial seizures than men [10]. Other studies suggest there may be biological differences to the severity of NCC-related seizures between men and women. A study in Mexico found that female sex is a risk factor for the severity of inflammatory response within brain parenchyma to a parasitic disease and that there is potentially some difference in immune response by sex to this organism [18].

Whether differences of prevalence, severity or risk between men and women are biological (related to hormones or chromosomes for example) or gendered (related to social, economic or other constructed factors) is unknown. Findings from different studies are often contradictory. For instance, a study of NCC hospitalized individuals in California found that men were more likely to have a lengthy hospital stays (>4 days) as compared to women and the severity of NCC appeared associated with male sex [16]. Yet, a previous study in Mexico found that Cysticercotic encephalitis was more frequent among young women [17].

It is thus important to start exploring the gendered dimensions of such differences in prevalence, severity and/or manifestation of NCC-related seizures. Gender differences are sometimes hidden in epidemiological data: for example, differential infection rates between women and men can be a result of exposures through occupation and other gendered divisions of space and activities that render different risks. [19]. Similarly, suffering from NCC can be experienced differently by women and men as they inhabit different spaces and social roles at various points across their life cycle. There is scant data on the experience and response to NCC through a gendered lens.

Our study collected qualitative data from people affected by seizures in rural Peru. This paper explores the different ways in which men and women explain and cope with the condition strongly associated with NCC in the area. Our analysis contributes to understanding the gendered differences of living with NCC and experiences of seizures. This paper draws from anthropological literature that defines the therapeutic process as the actions taken vis-à-vis an event recognized as a health problem [20]. In particular, this paper shows that gender norms shape the therapeutic process: who did what when the health problem began, what steps were taken during the search for a diagnosis, who decided what actions to be taken in response to the illness episode? [20]. The narratives shared by the participants affected by epilepsy also show that the “logics of care” are framed by structural factors such as poverty and a health system with limited capacity to respond to a chronic condition. Logics of care are the forms of care thought by the patient, their relatives and friends as more appropriate for diagnosing and treating the disease [21].

Our study shows the relevance of using a biopsychosocial approach to fully understand the implications of living with epilepsy in a rural area of Peru. The biopsychosocial approach recognizes that an ailment is not merely a physical problem, but it is also a subjective experience, socially produced and interpreted. The biopsychosocial approach enables us to move beyond a purely clinical understanding of a chronic condition such as epilepsy, in order to integrate biological, psychological and social aspects of being ill. It stresses that the interpretation of a condition is filtered through cultural understandings of disease and diagnosis, which in turn influences the treatment selection and the assessment of the treatment outcome [22].

## Methods

### Ethics statement

Ethics approval for the study was obtained from the Universidad Peruana Cayetano Heredia (approval No. 393-22-16, dated 16th Ocotber 2016).

We conducted a qualitative study that involved interviews with patients reporting epilepsy (n = 5), caregivers (n = 4) and 12 focus groups (FG) divided by gender from two rural communities in Piura, Peru. Where focus groups were not possible, individual interviews were conducted. Patients and caregivers were asked about their personal disease experience while focus group discussions probed about personal and second hand experience of the disease.

Data collection was conducted between February 2nd and February 21st 2017 in one single stretch. Those responsible for data collection had a bachelor’s degree in anthropology or sociology (2 men and 2 women), had previous experience collecting qualitative data in rural settings, and received an additional eight hours of training before beginning the fieldwork. Community members were invited to participate in the FG at community venues such as the community assembly. To identify people with NCC, fieldworkers asked local health workers and community members to identify participants known to be suffering from seizures. The other participants were contacted through snowball sampling. They had to be over 18 years of age and be able to give consent. After patients were interviewed, caregivers were invited to participate at their homes.

The interviews and focus group discussions covered a wide variety of topics described elsewhere [23]. In this paper we focus on responses to the following questions:

1. Focus Group Discussions: How does living with NCC affect a person?
2. Individual interviews to patients: How did you learn about your condition? How has living with epilepsy/seizures affected you? How has living with epilepsy/seizures affected your family?
3. Individual interviews to caregivers: How has living with somebody with epilepsy/seizures affected you? How has living with somebody with epilepsy/seizures affected your family?

To better understand the local context, fieldworkers also kept field notes describing the places or recording informal conversations.

All interviews were audiotaped, transcribed verbatim and coded using Atlas-ti. One person was in charge of coding all interviews with regular revisions from the PI (first author) and the research assistant. Codes were created a-priori. For this manuscript we used the following codes (Table 1). We additionally searched in each of the coded excerpts, for information relevant to identify gendered dimensions of the condition and its implications.

**Table 1:** Codes used for data analysis regarding Neurocysticercosis.

CODE			DEFINITION
**E. Neurocysticercosis/Seizures**			Use this code to capture any mention of the NCC / Seizures that cannot be captured in the sub codes.
**E1. Local terms**			It refers to the different words, terms or phrases used locally to refer to the NCC / Seizures
**E2. Causes / Explanations**			It refers to the reasons that participants provide to explain why a person develops NCC / Seizures
**E3. Diagnosis**			It refers to the actions that led the patient to know that he / she suffers from NCC / Seizures
**E4.**	**Impact of NCC**		It refers to the consequences that the disease has generated in the person, the home (family) and in the community around the NCC / Convulsions
	e4.1.	Individual Impact	It refers to the consequences that having the disease has generated in the person around the NCC.
	e4.2.	Family Impact	It refers to the consequences that the disease has generated in the family (household) around the NCC.
	e4.3.	Community Impact	It refers to the consequences that the disease has generated in the community around the NCC.
**E5. Treatment / Management of condition**			It refers to the places, people or practices that people implement to treat the NCC / Convulsions. It may include actions recommended by the doctor, up to the consumption of home remedies for this purpose.
**E6. Pork consumption**			Any mention of the consumption of pork, the raising of pigs for human consumption, the handling of pork with trichina, accessibility to consume it (e.g. who, frequency)

### Setting

**Fig 2:**
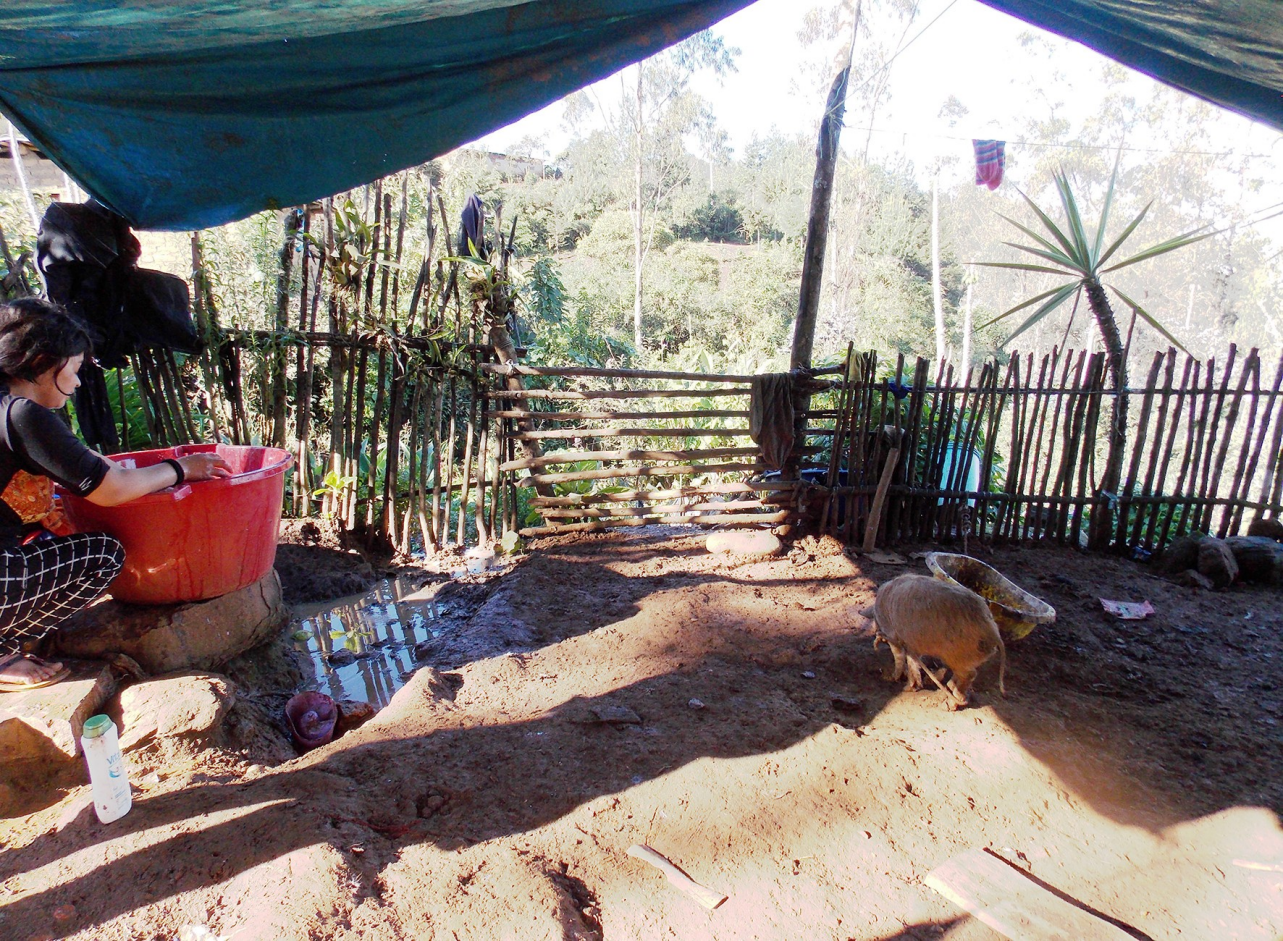
Fenced pigs in a house.

The study was conducted in two districts from the northern Peruvian region of Piura: Montero and Ayabaca. Water in the selected communities is obtained from small streams (Quebrada Chonta and Quebrada Ayabaca) or from non-treated piped water. Around 60% of families have borehole latrines in the backyard yet, most people practice open field defecation. Despite houses having corrals for their animals (See Fig 2), pigs are rarely corralled during the day, and the majority of them feed freely throughout the village and fields where they easily eat human faeces that might have tapeworm eggs. The adult tapeworm produce eggs that are released in the faeces of the human carrier [1]. Their stools containing tapeworm eggs then infect other pigs when they consume human faeces or other humans due to insufficient care in washing their hands after defecation [24]. Pigs are mostly fed by men and they are raised for sale. Some are consumed by villagers on festival days where eating poorly-cooked pork with cysts can infect the small intestine of humans [4,11]. Pigs are an economic asset and sometimes even when people see signs of cysticercosis *(*locally known as *“triquina”)* they will eat it and/or sell it for others to eat.

Once inside the pig or human, the eggs mature into larvae, which invade the intestinal wall, muscle, brain, and other tissues, causing cysticercosis which is usually asymptomatic [24]. When cysticercosis is located in the brain, it affects the central nervous system, manifesting itself through chronic headache, blindness, seizures (epilepsy if recurrent), hydrocephalus, meningitis, dementia and other symptoms [25].

## Results

### Participants

We interviewed five people affected by epilepsy (1 male) and four caregivers (1 male). Table 2 presents some of their sociodemographic characteristics. Three of the four women with epilepsy were housewives and performed agricultural activities, the other one worked as a cleaning lady at the Municipality. Two reported having suffered from gender violence which they associated with the onset of the epilepsy.

**Table 2:** Characteristics of patients with Epilepsy.

Pseudonym	Age	Gender	Marital Status	Education	Occupation
Laura	36	F	Single	Incomplete Technical Degree	Works as a cleaning lady at the Municipality
Cesar	36	M	Single	Technical Degree	Unemployed
Jimena	51	F	Married	3 years of Primary Education	Housewife/ Occasionally sells agricultural products from her land.
Aurora	50	F	Civil Union	5 years of Primary Education	Housewife/Agricultural activities
Carola	85	F	Married	Illiterate	Housewife

Three focus groups were male-only and nine were female-only. They had an average number of 6.8 participants and lasted an average of 51 minutes. Additionally, eight women and 20 men were interviewed individually using the same guide as for the FGD. Table 3 summarizes the number of participants by sex.

**Table 3:** Participants’ characteristics by sex.

Type of participant	Tool	Men	Women	Total
Patient with epilepsy	Individual interviews	1	4	5
Caregiver		1	3	4
Community Member		19	8	27
	Focus Groups	3 focus groupsn = 23	9 focus groups n = 59	82
**Total**				118

### Diagnosis

The following paragraphs describe the trajectories and therapeutic processes followed by the five patients of our study: Laura, Cesar, Aurora, Jimena, and Carola when they first experienced seizures. We will present the various interpretations of their condition and their initial care-seeking behaviours. As we will see, only the women remembered the time of diagnosis and the steps they (or their family members) took at that point in time. Cesar was a child when he first experienced seizures. Out of the five participants, only Aurora had been diagnosed with NCC, yet, it is highly probable that all women with seizures had NCC since the late on-set of epilepsy is associated with NCC [26].

### Laura: Epilepsy and menarche

At the age of thirteen, Laura suddenly started to experience blurred vision, itchy nose and sudden chills. She also remembers she would constantly yawn and sometimes, when people talked to her she “*drifted away*”. She remembers feeling “*like a statue*” that could not even blink. She was taken to the health facility in Ayabaca (District’s capital) where she was told seizures were a result of her having her period for the first time. Six years later, at the age of nineteen, Laura had seizures again and foamed at the mouth. She was taken to the health facility in Ayabaca where she was diagnosed with epilepsy. This diagnosis was confirmed in Lima at the age of 23 after the doctor performed several tests such as tomography, measurement of her blood pressure, blood tests and eye examinations. Laura did not know why she had epilepsy but she mentioned that one of her aunts had it too so maybe there was a hereditary factor in her condition. Her mother mentioned Laura was molested by a teacher while she studied to become a nurse technician and this experience also triggered her seizures.

### Aurora: Pork consumption

Aurora had her first seizures two years before the interview, when she was 34. She remembered she was at her daughter’s school meeting when suddenly she fell to the floor while convulsing. She first went to the local PHC facility where no diagnosis was provided; they just “stabilized” her. Later, her older children took her to Chiclayo (a city eight hours away from her community) where she was diagnosed with NCC. She was told that the origin of her condition was because she ate pork with “*triquina*” (cysticercosis). She is the only participant with NCC diagnosis.

### Jimena: Concussion, menopause and pork

Jimena told us that one night seven years ago (when she was 44 years old) she began to convulse while sleeping. Her husband, who was sleeping next to her ran to town asking for help and was told to give her *Toronjil* (lemon balm water). Jimena could not remember much about that night, but the following day she went to the local PHC where they prescribed her some medications. However, out of fear they might do more harm than good she did not follow the doctor’s instructions. Rather, she went to several traditional healers in four different towns. These healers diagnosed her with “*susto*” and “*daño*” caused by a neighbour. Both “*Susto*” and “daño" are culturally-bound syndromes; “susto” is the result of a negative emotional experience [27] and “*daño*” is a local way of referring to the “evil eye” or witchcraft. Traditional healers prescribed different preparations such as “*agua de oro*” (gold water), but they did not appear to work. A month later while she was cooking, she had seizures again. Jimena provided multiple explanations for her condition such as: a strong concussion, early menopause, and probably as a result of eating pork since her pigs were found to have *triquina* (cysticercosis).

### Carola: Domestic violence and *“debilidad”*

Carola did not remember the first time she experienced seizures so her husband, Don Carlos told us how it all started. He said that one day the year before, Carola (84 years old) was sitting outside her house and suddenly had a seizure. He held her and tended her but far from stopping she had another seizure. That day, she had four attacks in a row. People came and helped. Don Carlos called his daughter who lives in another town and asked her to take Carola with her. They agreed to meet in the town of Sullana. During the road trip Carola had another attack. The other passengers stopped a motorcycle that was driving by and asked the driver to take her as fast as he could to the Montero Health Centre where she was given a shot and referred to the nearest hospital in Sullana. Unfortunately, because she did not have a national identification card she was ineligible to use the national health insurance plan. By then, her daughter had reached Sullana and after being discharged, took her to her house in another city. Carola stayed 15 days with her daughter who took her to see a doctor and bought medications. Although Carola’s daughter told her she would take care of her, Carola decided to return to her house because there was no one to cook for her son. Carola did not know what condition she had, but she associated her seizures with “*mal aire*” and “weakness” as a result of a pregnancy with twins and domestic violence during her first marriage: “*My husband was*, *you can say*, *a little bit mean*. *He will start drinking and [hit you]*.*”* Don Carlos confirmed this information: “*her seizures are also the result of being weak*, *not the other way round*. *When she had her first husband she suffered lots of sadness*, *instead of eating food she ate punches…*”. Carola recalled feeling anger, grief and a constant desire to cry during her first marriage.

Carola told us that the first time she experienced seizures Don Carlos decided against taking her to a health facility immediately because it was the harvest season and he could not leave the town to take her. His precarious land tenure meant he could not delay the harvest (and get in debt with the landowner), and consequently his wife did not receive timely medical intervention.

### Cesar: God’s will

Cesar was the only patient who had his condition since he was a child. His parents told him he started having seizures at the age of two, then the seizures disappeared and re-appeared at the age of six, but was diagnosed at the age of seven in a hospital in Lima, where they did tests on his heart and brain.

All these stories illustrate the limited knowledge the patients and their families had about their condition and hint at the limited information they received from healthcare providers. This often happens in rural areas of Peru where health professionals underestimate the capacity of peasants to understand the diseases that affect them [28].

Another important thing to note is that two participants (Aurora and Cesar) were diagnosed in big cities (Chiclayo and Lima, each located eight and sixteen hours away from Piura by road) were their family members took them. This means the family had the financial ability to pay not only for the travelling expenses but also for the medical services received. Laura and Jimena were diagnosed in the nearest Health Centre in the town of Ayabaca which albeit closer, also entails spending money to travel. These expenses are not covered by the national health insurance. This shows that getting a diagnosis is linked to the family’s financial capacity to taking the patient to a health facility located away from the patient´s community where there is only a PHC facility.

In all cases, several years passed before receiving a diagnosis. This has to do with lack of access to biomedical information about what to do when a seizure occurs, having local explanations and traditional treatments in which they trust, and limitations in the health system. Health workers are not well trained in this condition and the nearest Health Centre in the area does not have a CT scan machine which is the only way to establish a definitive NCC diagnosis for those experiencing seizures.

### Living with seizures

All patients reported that living with NCC implied living in fear of having a seizure. Seizures were described as episodes where they “blacked out”, ran out of breath, or had “agitated” heartbeat. Community members described that people affected by NCC usually have strong headaches, feel dizzy and can even “lose their minds” (“*se vuelven como locos*”). Most importantly, FG participants stated those with NCC cannot lead a full, “normal” life because they are constantly tired, have migraines, and live in fear of having a seizure: “At any moment the drunkenness comes and the person cannot live in peace” (Woman in FG1).

**Fig 3:**
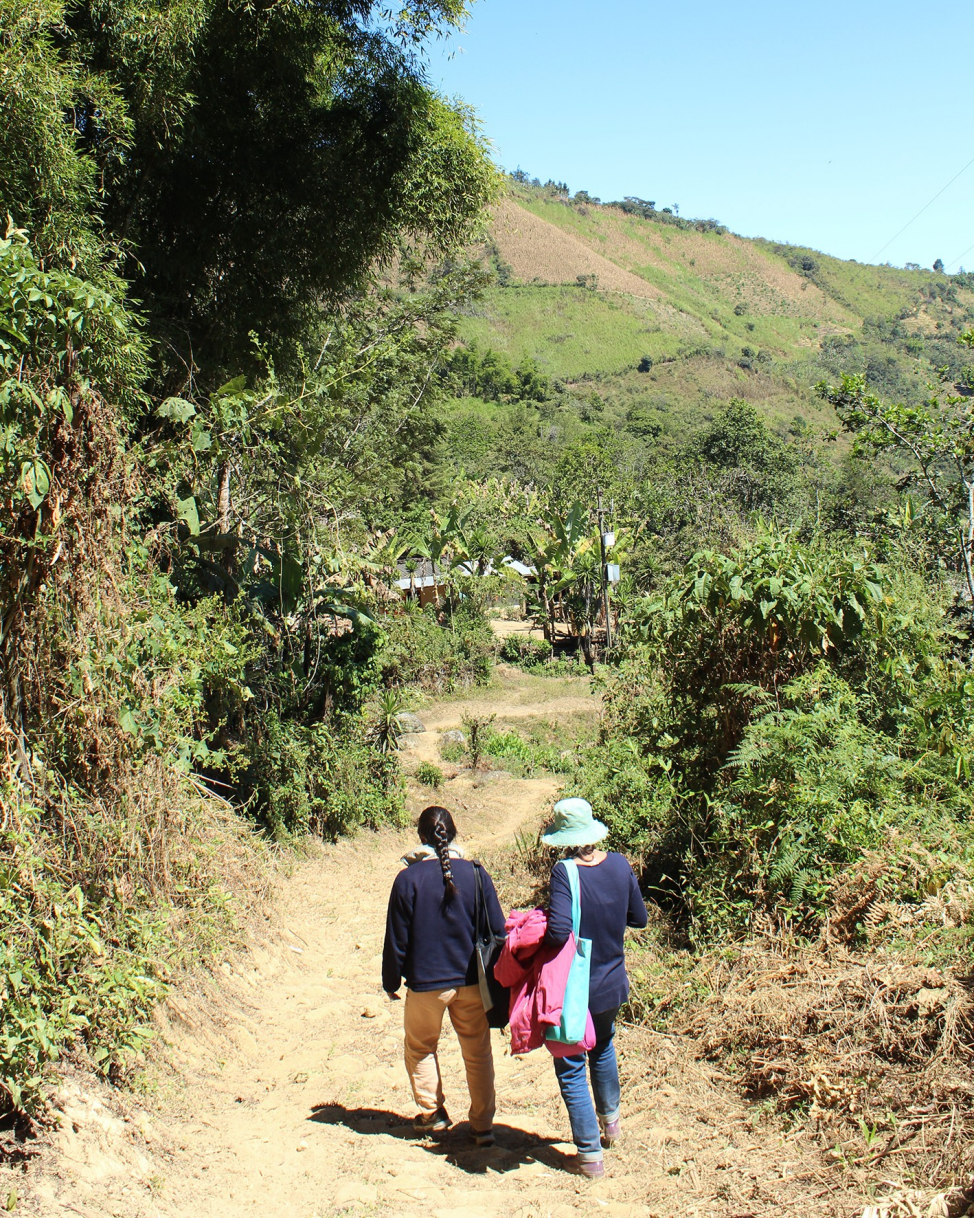
Rural Roads.

For women, being prone to unexpected seizures meant they could no longer engage in leisure activities such as weaving or knitting, or fully participate in domestic chores. They could not go walking by themselves to the fields because of fear of falling on their way there. Falling in this area is particularly dangerous since “roads” are narrow mountain paths along ledges with very little human traffic (albeit used for taking cattle to and from the fields), see Fig 3.

Most importantly women complained that they could no longer cook for their families either because as one woman said “*everything tastes sour*” or because they fear they might experience a seizure and fall into the woodstove and burn badly. Not being able to perform their household responsibilities meant others assumed this unpaid work. Women lamented and regretted burdening other family members such as their husbands or daughters who in two cases had to take over cooking and other domestic chores. In one case, the teenage daughter who took care of her mother had to postpone her post-secondary studies. Leonor said her mother’s illness worried her so much that she developed gastritis:

“When my mother started having seizures I got very stressed; I worried all the time. I remember sitting in the evenings and crying. My school grades went down, they were no longer good, because I was the top student in my class (…) but my mother could no longer do housework and I had to cook, go to school with all those worries. Sometimes I did not eat because I had to do my homework (and the house chores). As you know, a mother is indispensable in the house, she stays at home, makes lunch.” (Leonor, 18 years old)

Cesar, Aurora and Carola mentioned that they had introduced dietary changes as a result of their condition: avoiding pork or “*eating on time*” which means that you should not allow too much time in-between meals. FG participants frequently mentioned several dietary restrictions that people with epilepsy should follow, specifically abstaining from sweets, turkey, fish and pork. Interestingly, Cesar and female participants from two focus groups mentioned alcohol was also not allowed for people with epilepsy and highlighted the social implications of precluding men with epilepsy from consuming alcohol. Alcohol consumption in rural Northern Peru is a common practice on weekends and festivities, especially among men, and it is a behaviour associated with masculine identity. Thus, not being able to drink was seen as a difficult behavioural change for men.

Male respondents expressed concern about their reduced capacity to practice sports and perform income-generating activities if they had epilepsy. FG participants mentioned the case of a schoolteacher who, because of the regularity and intensity of his headaches, had to stop working. Sadly, this man eventually died without ever receiving a diagnosis. They also mentioned the case of a man who owned a passenger truck to transport people to and from communities to the city of Piura. People no longer wanted to ride in his truck because they feared that he might experience a seizure or have an anger fit (also associated with NCC) while driving.

Finally patients, caregivers and FG participants mentioned that a major problem for people with NCC was that they had to rely on medications: “*People have to take pills all the time*, *their life revolves around taking their medications*” (Male participant, FG40). Taking medications regularly was seen as major disruption not only because of the treatment itself but because of the financial costs. Medications are not always available at the PHC and people have to either travel to the nearest city and cover their own transportation expenses, or pay at a private pharmacy or clinic in the city.

## Discussion

Studies on gender and health have established that gender influences exposure to infection and risk of disease [29]. They have also established the complex interaction between exposure and biological susceptibility, probability of infection, severity of disease, and treatment outcomes. Our data does not allow us to make these connections (more exposure to the disease or more severity) but it points to the way in which gender norms frame therapeutic processes which include making decisions that enable timely diagnosis and treatment. Both diagnosis and treatment can significantly affect the quality of life of people with epilepsy. Our study shows that gender norms can shape access to care and use of available health services, both processes are also influenced by the socioeconomic status of patients.

The stories of Aurora, Jimena and Carola, show that it was either their husbands or their adult (often male) children who held decision making power around accessing local or distant medical facilities. Doyal’s study on gender and the political economy of health [30], discusses that men usually control the flow of financial resources within the household. They are also the ones in charge of making decisions around health expenditures for individual members of the household, playing a key role in women’s health status and position within their household. For instance, studies on decisions around obstetric emergencies in rural Peru and elsewhere have documented the prominent role of male members of a family in key decisions when delivery complications started [31,32]. Such studies point to the importance of acknowledging the figures of authority within households to understand health-related decisions. This is particularly relevant for NTDs such as NCC that can be easily prevented at an early stage and that, if gone undiagnosed and untreated, can have terrible chronic effects. Furthermore, the gendered division of labour, specifically pig-raising appear to reflect the agro-political economy of the household that impacts men and women’s health differently.

The therapeutic decisions described in this paper ought to be understood vis-à-vis poverty and medical pluralism. Poverty clearly limited patient’s choices when seizures started. For instance, Jimena could not have an immediate evacuation due to her family’s lack of financial resources, and Carola could not access health services in Sullana as a consequence of lack of proper identification documents. Carola is one of the thousands of women in Peru who do not have a national identification card [33] which in turn, expresses structural gender inequalities for older rural women.

Medical pluralism understood as the co-existence of various medical traditions, allowed patients to try different therapeutic options that appeared as more reliable to them or made more sense based on their understanding of the etiology of their seizures. Jimena went to several traditional healers in search for a diagnosis and a cure, even after having seen a health professional and Cesar’s parents did the same. The prevalent use of traditional medicine to treat seizures in Northern Peru has recently been documented in a study among 266 people with epilepsy that found that sixty seven percent of them reported using traditional medicine for their condition [34].

Our study, confirms the relevance of using a biopsychosocial model to understand the gendered nature of explanatory models of seizures. Carola established a causal connection between seizures and domestic violence and reproductive issues (having too many children) while Aurora was told the epilepsy onset might be related with menarche and her mother thought it could be related to an experience of sexual harassment by a teacher. Their social vulnerability is at the root of distressing experiences that, women in the selected communities attribute as the cause of epileptic symptoms. Associations between women´s distress and health conditions have been found in other studies in Latin America [35–37]. In rural Peru, Oths [38] described a syndrome called “*debilidad*.” Oths explains that *debilidad*, as other culture-bound syndromes tend to concentrate on women because in one way or another they are associated with their reproductive life and are also the embodiment of the everyday hardships and stress experienced by poor women. The explanations that women in our study give to their seizures shows the relevance of looking at other aspects of women’s lives [39]: their structurally subordinated position in society, difficult reproductive lives and their exposure to domestic violence, which is quite extended in the region, where a national survey found that between 13% and 52% of women had experienced intimate partner violence on the 12 months previous to the survey [40].

A study by Auditeau et al.[34] in a similar rural area of Peru found that “Emotional problems” (stress, anxiety, anger, family problems) were cited as the main reasons for epilepsy followed by neurocysticercosis (17.9%) It is also important to note that nearly 10% of patients in her study, mentioned witchcraft and spirits [34], which was also an explanation provided by Cesar´s parents and Jimena. Local explanatory models are relevant to understand not only the interpretation of symptoms but also the potential health-seeking behaviours and treatment options [19]. Explanatory models are context-specific, culturally shaped explanations of illness that frame the perceptions, experience and coping strategies [41].

The accounts of our participants also allow us to understand the complexity of epilepsy. From a biological/physical perspective having epilepsy implies, seizures and headaches which in turn, translates into psychological suffering such as living in fear of having a seizure at any moment, or feeling frustrated about their condition or (in the case of family members) feeling worried about their relative with epilepsy. The social consequences of having a debilitating condition such as NCC are also divided along gender lines. In the case of men, having epilepsy implies no longer being able to participate in income-generating activities as well as not being able to consume alcohol, an important socializing masculine behaviour in the area. Among women, seizures prevent them from performing their everyday household activities thus burdening other family members. Not being able to care for others in places like northern Peru where the gender norms strongly connect to the roles of women as caregivers in the family pose a significant additional emotional burden for women NCC patients. Mendenhall (2016) found similar feelings among Mexican women with diabetes. Women experienced a social tension between the social expectations of them as mothers and wives and what they could do due to their health problems [42]. As she explains: “cultural models of gendered behaviour place women at the centre of the home, as nurturer, caregiver, and food-preparer” [42:67] and these roles are disrupted by a disabling chronic condition such as epilepsy. From our informants’ accounts it appears that NCC is disrupting this important aspect of their lives and identity and further research should examine this.

### Directions for future research

Scholars can build on this work in three ways. First, understanding that the implications of any condition but particularly a chronic one ought to be studied not only along gender lines but also through an intersectionality lens to include conditions of poverty, geography, economic resources, ethnicity, and age among them. Second, NTDs such as NCC remain poorly diagnosed in rural areas and lay explanatory models are therefore employed to make sense and live with the impact of seizures. Finally, methodologically, this research reiterates the importance of using qualitative approaches to better understand not only the implications of living with a debilitating condition such as NCC, but the role of gender in understanding decision-making, experience and vulnerabilities.

Future studies could explore in detail how causal explanations of seizures, for example domestic violence, may be delaying access to care. Studies in similar contexts have found that oftentimes women with caring responsibilities within the home, face challenges in making time to seek healthcare in a timely fashion [27]. Another important research line would be to analyse the differential use of sanitary facilities which could help us understand the gendered risks of NCC infection.

A yet under-explored area of further gendered research concerns the practices of pig husbandry and free roaming of pigs among smallholder farmers where division of labour, time-use, space and the slaughter, preparation and consumption of pork meat may expose women and men differently at identifiable moments in the cycle of transmission. Further anthropological fieldwork with a gendered lens would likely pinpoint intersections of modifiable risk with improved human-animal environment that would benefit health of women and men.
